# Induction- and conditioning-protocol dependent involvement of NR2B-containing NMDA receptors in synaptic potentiation and contextual fear memory in the hippocampal CA1 region of rats

**DOI:** 10.1186/1756-6606-1-9

**Published:** 2008-09-30

**Authors:** Xue-Han Zhang, Long-Jun Wu, Bo Gong, Ming Ren, Bao-Ming Li, Min Zhuo

**Affiliations:** 1 Institute of Neurobiology and State Key Laboratory of Medical Neurobiology, Institutes of Brain Science, Fudan University, Shanghai 200032, PR China; 2 Department of Physiology, Faculty of Medicine, University of Toronto, 1 King's College Circle, Toronto, Ontario M5S 1A8, Canada

## Abstract

Long-term potentiation (LTP) in the hippocampal CA1 region requires the activation of N-methyl-D-aspartate receptors (NMDARs). Studies using genetic and pharmacological approaches have reported inconsistent results of the requirement of NR2B-containing NMDARs in LTP in the CA1 region. Pharmacological studies showed that NR2B-containing NMDARs are not required for LTP, while genetic studies reported that over-expression of NR2B-NMDARs enhances LTP and hippocampus-dependent memory. Here, we provide evidence showing that the functional role of NR2B-NMDARs in hippocampal LTP and memory depends on LTP-inducing and behavior-conditioning protocols. Inhibition of NR2B-NMDARs with the NR2B selective antagonist ifenprodil or Ro25-6981 suppressed LTP induced by spike-timing protocol, with no impact on LTP induced by pairing protocol or two-train high-frequency stimulation (HFS) protocol. Inhibition of NR2B-NMDARs did not affect the late phase LTP induced by four-train HFS. Ca^2+ ^imaging showed that there was difference in kinetics of intracellular Ca^2+ ^signals induced by spiking-timing and pairing protocols. Pre-training intra-CA1 infusion of ifenprodil or Ro25-6981 impaired the contextual fear memory induced by five CS-US pairings, with no effect on the memory induced by one CS-US pairing.

## Introduction

N-methyl-D-aspartate receptors (NMDARs) are critical for synaptic plasticity in the hippocampus and hippocampus-dependent learning and memory [1-3]. NMDAR is heteromer. Functional NMDAR has a combination of NR1 subunit and at least one of NR2 subunits (A-D). In the hippocampus, NR2A- and NR2B are two predominant NR2 subunits for NMDARs [4,5]. The NR2-subunit compositions of NMDAR, whether they are NR2A- or NR2B-containing, determines the pharmacological and kinetic properties of the NMDAR-mediated currents [6-8]. Studies have suggested that NR2A- *vs*. NR2B-NMDARs may mediate distinct synaptic functions [9,10].

Synaptic long-term potentiation (LTP) has been widely accepted as a synaptic mechanism for learning, memory and other higher-order brain functions [1,2]. LTP can be induced in brain-slice preparations by different induction protocols [1,11]. A recent study by Wang's group reported that NR2A- and NR2B-NMDARs may be differentially involved in synaptic plasticity in the CA1 region, with LTP being mediated by NR2A-NMDAR and LTD by NR2B-NMDAR [9]. However, the conclusion that NR2B-NMDAR is selectively involved in the induction of LTD but not LTP has been challenged by the studies using NR2A-knockout mice [12,13] and over-expression of NR2B-NMDARs [14], suggesting the existence of NR2A-independent forms of LTP. Furthermore, it should be noted that these previous studies used different slice preparations and, especially, different protocols to induce LTP. It might be possible that NR2B-NMDARs contribute differentially to LTP induced under different conditions.

Hippocampus is an essential structure for flexible spatial memory and contextual fear memory. For example, inactivation of or lesion to the hippocampal CA1 region results in a severe deficit in contextual fear memory [15,16]. It is well known that NMDARs in the CA1 region are involved not only in memory acquisition [3,17,18], but in memory retrieval as well [19]. These previous studies used non-selective NMDAR antagonists like MK-801 to access the contributions of hippocamopal NMDARs to memory acquisition and retrieval. The role of NR2B-NMDAR has been largely unclear and remains to be established.

In the present study, we examined the effects of selective blockade of NR2B-NMDARs on LTP induced by different protocols including spike-timing protocol [20,21], paring protocol and high-frequency stimulation (HFS) protocol. We also investigated the intracellular calcium signals under different LTP-inducting protocols. We further addressed the impacts of intra-CA1 blockade of NR2B-NMDARs on the acquisition and retrieval of contextual fear memory induced by different conditioning strengths.

## Materials and methods

### Animals

Rats and mice were used for the present study. The animals housed in plastic cages and maintained on a 12-h light/dark cycle. Food and water were available *ad libitum *throughout the experiment. All experimental procedures were in accordance with the Guide for the Care and Use of Laboratory Animals issued by the National Institutes of Health, USA (1996) and approved by the Ethical Committee of Animal Experiments at Fudan University Institute of Neurobiology (Shanghai, China) and by the Animal Studies Committee at the University of Toronto (Toronto, Canada).

### Drugs

To address the functions of NR2B-containing NMDARs, we used two selective antagonists: the non-competitive NR2B antagonist ifenprodil tartrate salt (Sigma-Aldrich Co., USA) and its derivative Ro25-6981 hydrochloride (Tocris, UK). In LTP recording experiments, we used the 3 μM dose of ifenprodil and the 0.3, 0.5 or 3.0 μM doses of Ro25-6981 to block NR2B-NMDARs. In behavioral experiments, we locally infused the 0.2 μg dose of ifenprodil or the 5.0 μg dose of Ro25-6981 into the CA1 region to inhibit NR2B-NMDARs.

To dissect the functions of NR2A-containing NMDARs, the relatively selective NR2A antagonist NVP-AAM077 (a generous gift from Novartis Pharma, Basel, Switzerland) was used. We selected the 0.3 μM dose of NVP-AAM077 in LTP recording experiments to dissect NR2A-NMDAR contributions, as our previous study showed that NVP-AAM077 at the dose of 0.3 μM did not affect NR2B-NMDAR currents *in vitro *[22]. For behavioral experiments, we locally infused the 0.012 and 0.12 μg doses of NVP-AAM077 into the CA1 region to block NR2A-NMDARs. The 0.12 μg/μl dose of NVP-AAM077 is equal to the 0.2 μg/μl dose of ifenprodil in molarity.

### Whole-cell patch clamp recordings

Sprague-Dawley rats (male, 200–220 g, 8–10 week old) were used for the experiments of whole-cell patch clamp recordings. Rats were anesthetized with inhaled isoflurane. Transverse hippocampal slices (300 μm in thickness) were rapidly prepared. Slices were incubated at room temperature for at least 1 h, and were then transferred to a recording chamber. The chamber was perfused at a speed of 2 ml/min with artificial cerebrospinal fluid (ACSF) consisting of (in mM): 124 NaCl, 2.5 KCl, 2 CaCl_2_, 1 MgSO_4_, 25 NaHCO_3_, 1 NaH2PO_4_, and 10 glucose (saturated with 95% O_2 _and 5% CO_2_) and 100 μM picrotoxin. The CA3 region was removed in order to reduce transmission of picrotoxin-induced epileptiform bursting to the CA1 region. Whole-cell patch clamp recordings were made in CA1 pyramidal neurons. Targeted neurons were voltage clamped at -60 mV. EPSCs were evoked by extracellular stimulation of Schaffer collaterals using a bipolar stimulating electrode at a rate of 0.02 Hz. The stimulating electrode was placed at about 100 μm distant from the cell body.

Spike-timing and pairing protocols were used to induce LTP in patched cells. In spike-timing protocol, three presynaptic stimuli at 30 Hz, which caused three EPSPs, were paired with three postsynaptic action potentials (APs), and did so 15 times with an interval of 5 s. Presynaptic stimulus was delivered 10 ms before postsynaptic AP. The recording pipette (3–5 MΩ) was filled with solution containing (in mM): 145 K-gluconate, 5 NaCl, 1 MgCl_2_, 0.2 EGTA, 10 HEPES, 2 Mg-ATP, and 0.1 Na_3_-GTP (adjusted to pH 7.2 with KOH). In pairing protocol, a train of 200 stimulation pulses (at 2 Hz) were delivered presynaptically, paired with a depolarization (to -5.0 mV) of postsynaptic cell (patched cell). The recording pipette (3–5 MΩ) was filled with solution containing (in mM): 102 Cs-gluconate, 3.7 NaCl, 11 BAPTA, 0.2 EGTA, 20 HEPES, 2 Mg-ATP, 0.3 Na3-GTP and 5 QX-314 (adjusted to pH 7.2 with CsOH). After EPSC recording was stable for 10 min, spike-timing protocol or pairing protocol was used to induce LTP. The access resistance was 15–30 MΩ. The input resistance was monitored throughout the recording experiment. Data were discarded if input resistance changed by more than 20%.

### Field potential recordings

C57BL/6 mice (male, 6–8 week old) were used for the experiments of field-potential recordings. Mice were anesthetized with inhaled isoflurane. Transverse slices of hippocampus were rapidly prepared and maintained in an interface chamber at 28°C, in which the slices were perfused with ACSF. The slices were kept in the recording chamber for at least 2 h before the experiments. A bipolar tungsten stimulating electrode was placed in the stratum radiatum of the CA1 region, and extracellular field potentials were recorded in the stratum radiatum, using a glass microelectrode (3–12 MΩ, filled with ACSF). Stimulation intensity was adjusted to produce a response with 0.5~1.0 mV amplitude. Test responses were elicited at 0.02 Hz. Two trains of HFS (100 pulses at 100 Hz, with inter-train interval of 20 s) were used to induce early LTP (E-LTP) and four trains of HFS (100 pulses at 100 Hz, with inter-train interval of 5 min) to induce late LTP (L-LTP).

### Calcium Imaging

C57BL/6 mice (male, 3–4 week old) were used for the experiments of calcium imaging. Mice were anesthetized with inhaled isoflurane and were decapitated. Transverse slices of hippocampus (300 μm in thickness) were prepared and transferred to a submerged recovery chamber with oxygenated (95% O_2 _and 5% CO_2_) ACSF at room temperature. Oregon green BAPTA-1 (OGB-1, 0.4 mM; Molecular Probes) was dialyzed into hippocampal CA1 neurons by whole-cell patch pipettes. Once patched in whole-cell mode, the neurons were maintained for at least 10 min to allow for filling with OGB-1 before image acquisition. Fluorescent signals were imaged by a confocal microscope (Fluoview FV 1000, Olympus, Tokyo, Japan). The laser with a wavelength of 488 nm was used for excitation and fluorescence was recorded through a bandpass filter (500–550 nm). The images were acquired using a 40×, 0.8 numeric aperture water-immersion objectives every 0.5 s after a 0.188-s exposure to 488-nm light. XYT image galleries were collected. Average fluorescence intensity in the region of interests (ROI) was measured for quantification. The ROI was around 100 μm from soma. The intensity was expressed as F/F0, where F0 is the fluorescence intensity before LTP induction.

### Fear conditioning

Fear conditioning was performed in a plexiglas conditioning chamber with a metal grid floor (San Diego Instruments, USA). Infrared equipment was located on the walls to monitor freezing behavior of rats. Rats were given 5 min to acclimate to the chamber pre-conditioning. Two conditioning protocols were employed. For one CS-US pairing protocol, rats were presented with one tone (conditioned stimulus, CS; 2.2 kHz and 96 dB for 30 s), which co-terminated with a foot shock (unconditioned stimulus, US; 1.0 mA, 2 s). For five CS-US pairing protocol, rats received five CS-US pairings (1.0 mA, 0.5 s for each US), with inter-pairing interval of 90–120 s [23]. After conditioning, rats were placed back to home cages.

Memory retention was tested 48 h post-conditioning. For testing of contextual fear memory, rats were placed into the original chamber, where the rats had been conditioned, and allowed to stay there for 3 min without tone or footshock. Freezing response during this period was used as a measure for contextual fear memory. For testing of auditory fear memory, rats were placed into a novel chamber for 90 s and were then given three CSs, each lasting 30 s with inter-CS interval of 20 s. Freezing response during the CS presentations was used as a measure for auditory fear memory.

### Histology

To verify the locations of drug infusion, rats were anesthetized with pentobarbital sodium (50 mg/kg, i.p.) and perfused transcardially with saline, followed by 4% (vol/vol) formaldehyde solution. Rat brains were placed into 30% (wt/vol) sucrose solution and subsequently cut into 40~50 μm sections with a cryostat (Leica CM900, Germany). Brain sections were mounted on gelatin-subbed glass slides and stained with neural red (1% in ddH2O). Images were taken using a light microscope (Leica DMRXA Q5001W) equipped with a CCD camera.

### Data analysis

Data in the text and figures are expressed as means± SEM. Student's t-test was used to compare LTP data. An one-way Analysis of Variance (ANOVA) was employed to compare behavioral data, with planned comparisons as post hoc analysis. In all cases, p < 0.05 was considered significant. Statistical analysis was performed using STATISTICA (StatSoft Inc., Chicago, IL, USA).

## Results

### LTP induced by spike-timing protocol

**Figure 1:**
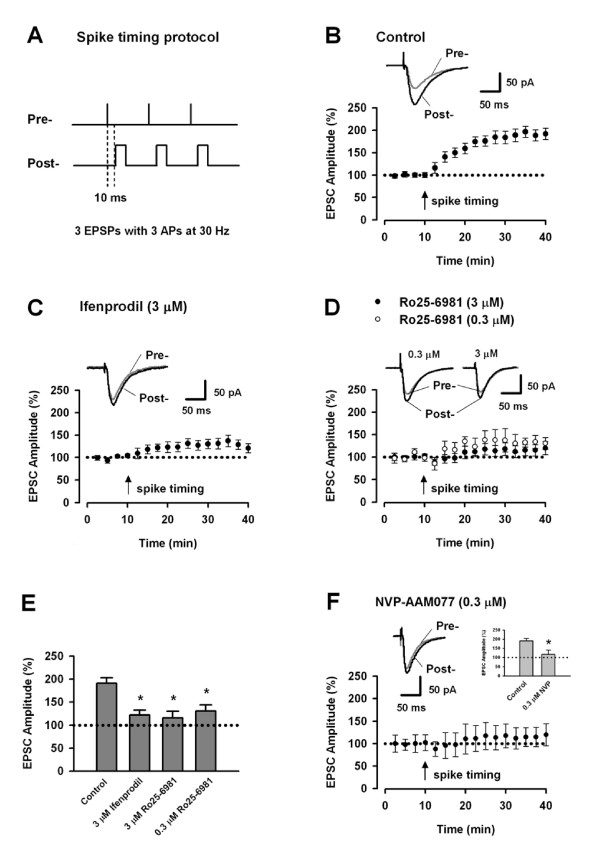
**NR2B-NMDARs are required for LTP induced by spike-timing protocol in area CA1**. A. Schematic diagram of the spike-timing protocol. B. Spike-timing protocol, as indicated by the arrow, induced a significant LTP in CA1 pyramidal neurons (n = 8). Sample traces of EPSC are the averages of 7 consecutive responses recorded during 5–10 min and 25–30 min, respectively. C. Bath application of ifenprodil partially blocked the LTP. n = 8 neurons D. Bath application of Ro25-6981 partially blocked the LTP. n = 7 neurons for 0.3 μM Ro25-6981; n = 8 neurons for 3 μM Ro25-6981. E. Histograms showing the effects of ifenprodil and Ro25-6981 on the LTP. *p < 0.05 vs. control. F. NR2A-NMDARs are required for LTP induced by spike-timing protocol in area CA1. Bath application of NVP-AAM077 blocked the LTP. n = 7 neurons. inset: Histogram showing the effect of NVP-AAM077 on the LTP. *p < 0.05 vs. control.

To test whether the involvement of NR2B-NMDARs is dependent on specific LTP induction paradigm, we first examined the role of NR2B- and NR2A-NMDARs in LTP induced by spike-timing protocol (also named EPSPs-APs protocol) (Figure 1A). To be consistent with the experimental conditions used by Wang's Group [9], we first performed whole-cell patch clamp recordings in CA1 pyramidal neurons from rat hippocampus. Spike-timing protocol caused a significant potentiation of synaptic responses (Figure 1B: 190.8 ± 12.2% of baseline at 25–30 min post-induction, n = 8 slices; *p *< 0.05 vs. baseline). We then tested the possible contribution of NR2B-NMDARs. The non-competitive, selective NR2B-NMDAR antagonist ifenprodil (3 μM) was perfused throughout the experiments. As shown in Figure 1C and 1E, ifenprodil significantly reduced the potentiation (121.4 ± 10.7% of baseline, n = 8 slices, *p *< 0.05 vs. control). Similar inhibitory effects were found with another NR2B-NMDAR antagonist Ro25-6981 (Figure 1D and 1E: For 0.3 μM Ro25-6981, 130.6 ± 13.1% of baseline, n = 7 slices, *p *< 0.05 vs. control; For 0.3 μM Ro25-6981, 116.1 ± 13.4% of baseline, n = 8 slices, *p *< 0.05 vs. control).

Bath application of the NR2A relatively selective antagonist NVP-AAM077 (0.3 μM) also reduced LTP induced by spike-timing protocol (Figure 1F: 108.9 ± 12.1% of baseline, n = 7 slices, *p *< 0.05 vs. control). These results provide evidence that both NR2B- and NR2A-NMDARs contribute to LTP induced by spike-timing protocol.

### LTP induced by pairing protocol

**Figure 2:**
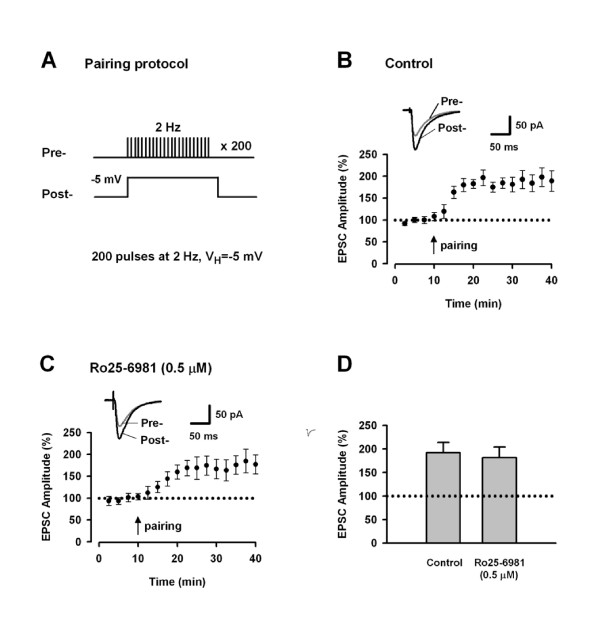
**NR2B-NMDARs are not required for LTP induced by pairing protocol in area CA1**. A. Schematic diagram of the pairing protocol. B. Pairing protocol, as indicated by the arrow, induced a significant LTP in CA1 pyramidal neurons (n = 6). Sample traces of EPSC are the averages of 7 consecutive responses recorded during 5–10 min and 25–30 min, respectively. C. Bath application of Ro25-6981 (0.5 μM) had no effect on the LTP (n = 5). D. Histogram showing the effect of Ro25-6981 on the LTP. p > 0.05 vs. control.

Next, we examined the effect of NR2B inhibition on LTP induced by pairing protocol (Figure 2A). Whole-cell recordings were done in CA1 pyramidal cells. The pairing protocol induced a significant potentiation of synaptic responses (Figure 2B: 192.1 ± 22.0% of baseline, n = 6 slices; *p *< 0.05 *vs*. baseline). Bath application of 0.5 μM Ro25-6981 did not affect the synaptic potentiation (Figure 2C: 181.6 ± 22.8% of baseline, n = 5 slices; *p *< 0.05 *vs*. baseline). As shown in Figure 2D, there was no significant difference in LTP amplitudes in the presence and absence of Ro25-6981 (25–30 min post-induction, *p *> 0.05 for Ro25-6981 *vs*. control). This result indicates that NR2B-NMDARs are not required for LTP induced by the pairing protocol in the CA1 region, consistent with the previous report by Liu et al. [9]

### LTP induced by high-frequency stimulation

**Figure 3:**
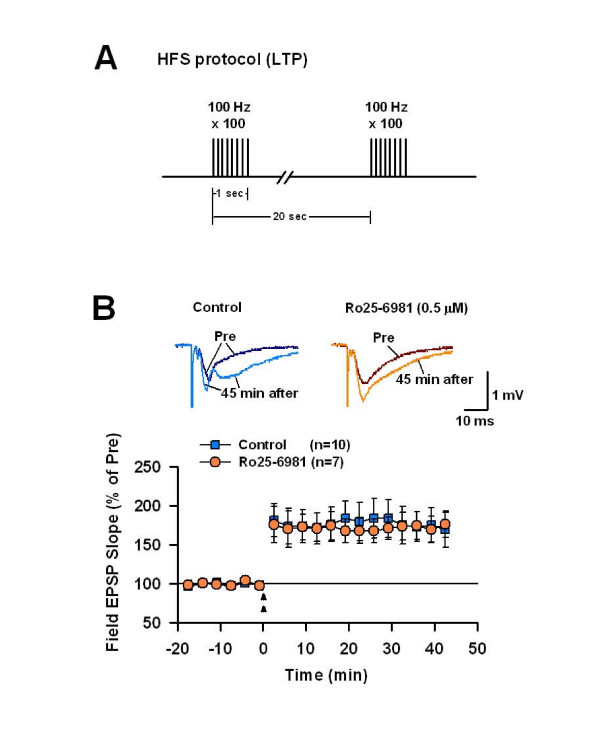
**NR2B-NMDARs are not required for LTP induced by HFS protocol in area CA1**. A. Schematic diagram of the high frequency stimulation (HFS; 2 train). B. LTP of field EPSP induced by the HFS in control (n = 10 slices) and in the presence of Ro25-6981 (n = 7 slices). Ro25-7981 had no effect on the LTP.

We then investigated the effect of NR2B-NMDAR blockade on LTP induced by HFS using *in vitro *field-potential recordings. A two-train HFS induced a robust and sustained potentiation of synaptic responses in the CA1 region in control experiments (Figure 3B: 174.2 ± 22.5% of baseline at 40–45 min post-HFS, *p *< 0.05 *vs*. baseline, n = 10 slices). A similar amount of potentiation was observed in the presence of 0.5 μM Ro25-6981 (Figure 3B: 172.3 ± 15.5% of baseline, *p *< 0.05 *vs*. baseline; *p *> 0.05 *vs*. control; n = 7 slices), indicating that LTP induced by HFS does not involve NR2B-NMDARs.

**Figure 4:**
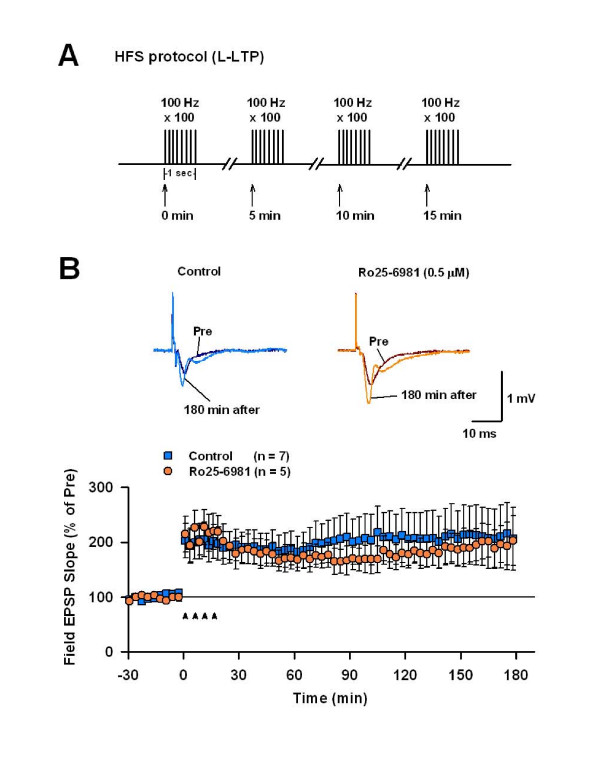
**NR2B-NMDARs are not required for L-LTP induced by HFS protocol in area CA1**. A. Schematic diagram of the high frequency stimulation (HFS; 4 trains). B. Late phase LTP of field EPSP induced by the HFS in control (n = 7 slices) and in the presence of Ro25-6981 (n = 5 slices). Ro25-7981 had no effect on the L-LTP.

**Figure 5:**
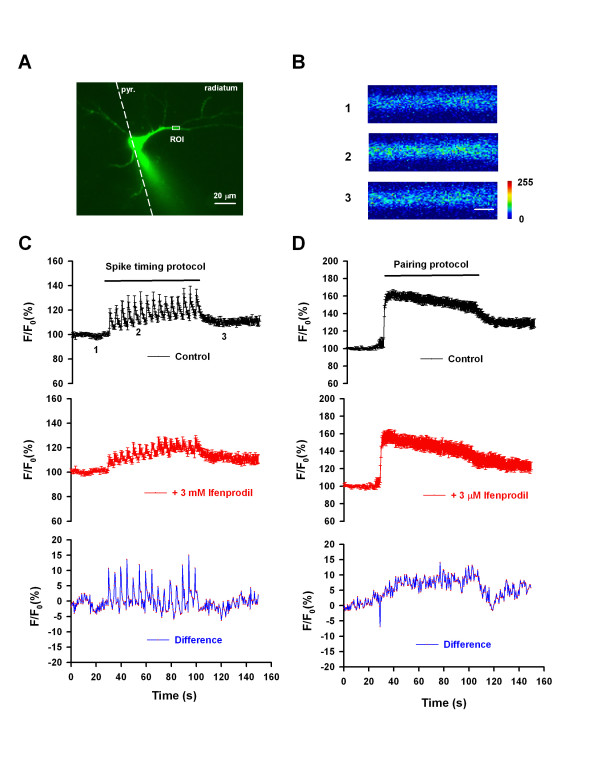
**NR2B-NMDAR mediated Ca^2+ ^influx under spiking-timing and pairing protocols**. A. A representative image showing the CA1 pyramidal neuron filled with OGB-1. ROI, region of interests. B. Raw sample fluorescence images from the ROI before (1), during (2) and after (3) induction of LTP with the spike-timing protocol. Scale bar, 1.0 μm. C. Elevation of Ca2+ signal in the ROI during induction of LTP with the spike-timing protocol (upper). Treatment with ifenprodil (3 μM) reduced the Ca2+ signal (middle). The difference of [Ca2+] signals in control and in the presence of ifenprodil (lower) shows the NR2B-NMDAR mediated Ca2+ influx. D. Elevation of Ca2+ signal in the ROI during induction of LTP with the pairing protocol (upper). Treatment with ifenprodil (3 μM) reduced the Ca2+ signal (middle). The difference of [Ca2+] signals in control and in the presence of ifenprodil (lower) shows the NR2B-NMDAR mediated Ca2+ influx.

To address the possible role of NR2B-NMDARs in L-LTP, we used a four-train HFS to induce L-LTP in the CA1 region (Figure 4A). As shown in Figure 5B, the potentiation of filed EPSP was robust and sustained for over 3 h after the delivery of the HFS (209.6 ± 57.0% of baseline at 170–180 min post-HFS *p *< 0.05 *vs*. baseline, n = 7 slices). Bath application of 0.5 μM Ro25-6981 did not affect the L-LTP (Figure 4B: 200.7 ± 33.5% of baseline; *p *< 0.05 *vs*. baseline; *p *> 0.05 *vs*. control, n = 5 slices). This result indicates that NR2B-NMDARs are not required for the L-LTP.

### Ca^2+ ^signals triggered by spike-timing and pairing protocols

Calcium influx into postsynaptic neurons is a critical event in synaptic plasticity in the CA1 region. Here, we examined the intracellular Ca^2+ ^signals elicited by spike-timing and pairing protocols and evaluated the contributions of NR2B-NMDARs using calcium imaging. The two protocols triggered an increase in [Ca^2+^]_i _with different kinetics (Figure 5C and 5D, *upper*). Treatment with ifenprodil (3 μM) reduced the Ca^2+ ^signals under both protocols (Figure 5C and 5D, *middle*). By calculating the difference of the signals in control and in the presence of ifenprodil, we obtained the NR2B-NMDAR mediated Ca^2+ ^signals (Figure 5C and 5D, *lower*). As shown, the NR2B-NMDAR mediated calcium signal under the spike-timing protocol was much different in kinetics but not in absolute quantity from that under the pairing protocol. This difference in kinetics of Ca^2+ ^efflux might help explain why NR2B-NMDARs are required for LTP induced by spike-timing protocol but not pairing protocol. It may be possible that the fluctuation of the NR2B-mediated Ca^2+ ^efflux is a critical factor for induction of LTP under the spike-timing protocol.

### Acquisition of contextual fear memory

A previous study in our laboratory showed that intra-CA1 blockade of NR2B-NMDARs with Ro25-6981 (5.0 μg) had no effect on the acquisition of contextual fear memory in both rats and mice [24]. In that study, we trained the animals with a single CS-US pairing. Here, we re-examined the effect of intra-CA1 blockade of NR2B-NMDARs on the acquisition of contextual fear memory using another selective NR2B-NMDAR antagonist ifenprodil. We also investigated the effect of intra-CA1 blockade of NR2A-NMDARs, using the relatively selective NR2A-NMDAR antagonist NVP-AAM077.

**Figure 6:**
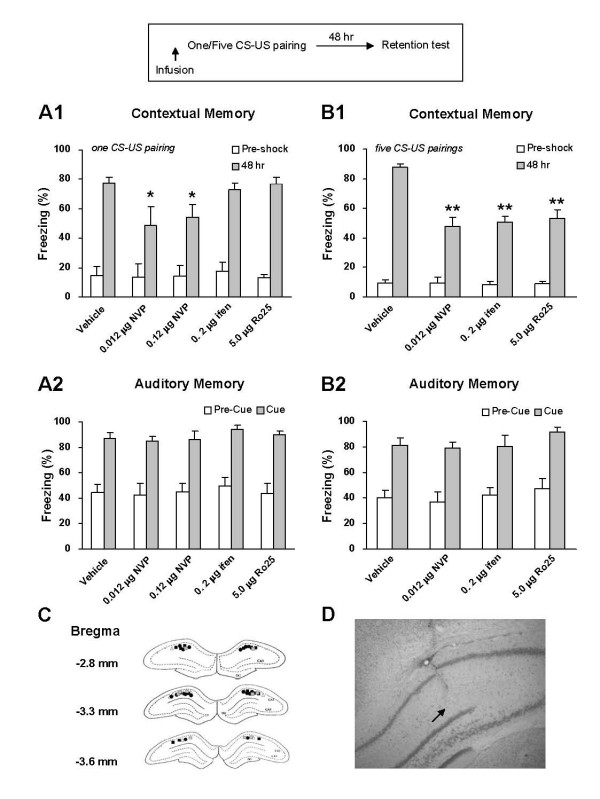
**NR2B-NMDARs are required for the acquisition of contextual fear memory induced by the five but not one CS-US pairing conditioning**. A. Pre-training intra-CA1 inhibition of NR2B-NMDARs had no impact on, while inhibition of NR2A-NMDARs impaired 48-h contextual fear memory induced by the single CS-US pairing protocol (A1). The acquisition of auditory fear memory was intact (A2). The result with Ro25-6981 was reported in the previous study (Zhao et al., 2005). *p < 0.05 *vs*. vehicle B. Pre-training intra-CA1 inhibition of NR2B- or NR2A-NMDARs impaired 48-h contextual fear memory induced by the five CS-US pairing protocol (B1). The acquisition of auditory fear memory was intact (B2). **p < 0.01 *vs*. vehicle. C. Reconstruction of the infusion sites in the CA1 region. Filled squares: vehicle; Open circles: 0.012 μg NVP-AAM077; Filled circles: 0.12 μg NVP-AAM077; Open triangles: ifenprodil; Grey squares: Ro25-6981. D. A representative coronal section showing an infusion site of ifenprodil in the CA1 region.

Intra-CA1 infusion of NVP-AAM077 (0.012 μg in 1 μl PBS, n = 6 rats; 0.12 μg in 1 μl PBS, n = 8 rats), ifenprodil (0.2 μg in 1 μl PBS, n = 7 rats), or Ro25-6981 (5.0 μg in 1 μl PBS, n = 8 rats) was performed 15 min before the animals received the single CS-US pairing protocol. PBS was similarly infused as vehicle control (1 μl; n = 9 rats). Fear memory was tested 48 h post-conditioning. As shown in Figure 6A1, rats with intra-CA1 infusion of ifenprodil demonstrated no difference in contextual freezing score in relative to vehicle controls (F(1,14) = 0.23, *p *> 0.05 for ifenprodil vs. vehicle). By contrary, rats with intra-CA1 infusion of NVP-AAM077 exhibited a severe deficit in contextual fear memory (F(1,13) = 7.93, *p *< 0.01 for 0.012 μg NVP-AAM077 vs. vehicle; F(1,15) = 6.13, *p *< 0.05 for 0.12 μg NVP-AAM077 vs. vehicle). Auditory fear memory was tested 1 h after contextual fear memory was tested. As shown in Figure 6A2, rats with intra-CA1 blockade of NR2B- and NR2A-NMDARs showed no deficit in auditory fear memory (F(4,31) = 0.60, *p *> 0.05). Thus, the acquisition of contextual fear memory induced by the single CS-US pairing involves NR2A- but not NR2B-NMDARs in the CA1 region.

Considering that the importance of NR2B-NMDARs for LTP induction is protocol-dependent, we attempted to know whether the role of NR2B-NMDARs in memory acquisition is also dependent on training paradigms. To address this, we examined the effects of intra-CA1 inhibition of NR2B- as well as NR2A-NMDARs on the acquisition of contextual fear memory induced by the five CS-US pairing conditioning. Our pilot experiment showed that the five CS-US pairing protocol could induce more freezing responses than the single CS-US pairing protocol did (Table 1).

**Table 1:** Contextual fear memory induced by the one and five CS-US pairing protocols

**Protocol**	**Pre-shock**	**48-h Retention Test**
Protocol 1: 1 CS-US (US: 1.0 mA, 2.0 s)	11.27 ± 4.65	72.86 ± 3.20
Protocol 2: 5 CS-US (US: 1.0 mA, 0.5 s)	9.53 ± 2.05	90.00 ± 1.77 **

Data is expressed as the percentage of freezing (mean ± SEM). Protocol-1: n = 7; Protocol-2: n = 6. **p < 0.01 vs. Protocol 1, *t*-test.

Intra-CA1 injection of NVP-AAM077 (0.012 μg in 1 μl PBS, n = 6 rats), ifenprodil (0.2 μg, n = 7 rats), or Ro25-6981 (5.0 μg, n = 8 rats) was performed 15 min before the animals was trained with the five CS-US pairing protocol. PBS was similarly infused as vehicle control (1 μl, n = 7 rats). Fear memory was tested 48 h post-conditioning. As shown in Figure 6B1, rats treated with NVP-AAM077, ifenprodil or Ro25-6981 showed a deficient contextual fear memory as compared with vehicle controls (F(1, 11) = 35.3, *p *< 0.01 for NVP-AAM077 vs. vehicle; F(1, 12) = 31.21, *p *< 0.01 for ifenprodil vs. vehicle; F(1, 13) = 26.05, *p *< 0.01 for Ro25-6981 vs. vehicle). However, the rats with intra-CA1 blockade of NR2B- and NR2A-NMDARs showed a normal auditory fear memory (Figure 6B2: F(3,24) = 0.72, *p *> 0.05). Thus, both NR2A- and NR2B-NMDARs in the CA1 region are required for the acquisition of contextual fear memory induced by the five CS-US pairing conditioning.

### Retrieval of contextual fear memory

As just described, NR2B-NMDARs in the CA1 region are involved in the acquisition of contextual fear memory in a conditioning-strength dependent way. Here, we wanted to know if NR2B-NMDARs are involved in the retrieval of contextual fear memory in a similar way.

**Figure 7:**
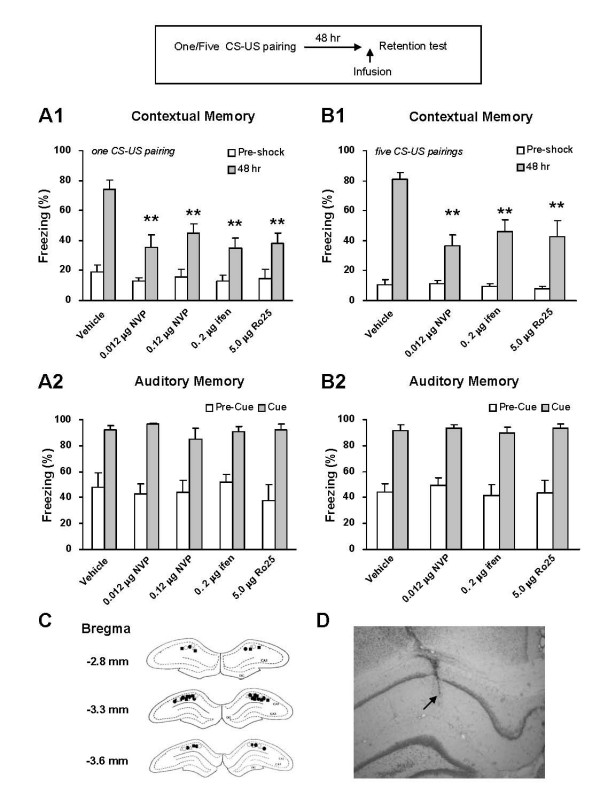
**NR2B-NMDARs are required for the retrieval of contextual fear memory induced by both five and one CS-US pairing conditioning**. A. Pre-retrieval intra-CA1 inhibition of NR2B- or NR2A-NMDARs impaired the expression of 48-h contextual fear memory induced by the single CS-US pairing protocol (A1). The expression of 48-h auditory fear memory was intact (A2). **p < 0.01 *vs*. vehicle. B. Pre-retrieval intra-CA1 inhibition of NR2B- or NR2A-NMDARs impaired the expression of 48-h contextual fear memory induced by the five CS-US pairing protocol (B1). The expression of 48-h auditory fear memory was intact (B2). **p < 0.01 *vs*. vehicle. C. Reconstruction of the infusion sites in the CA1 region. Filled squares: vehicle; Filled circles: NVP-AAM077; Open triangles: ifenprodil; Open circles: Ro25-6981. D. A representative coronal section showing an infusion site of ifenprodil in the CA1 region.

First, we examined the effects of intra-CA1 blockade of NR2B- as well as NR2A-NMDARs on memory retrieval for the single CS-US pair conditioning. NVP-AAM077 (0.012 μg in 1 μl PBS, n = 6 rats; 0.12 μg in 1 μl PBS, n = 7 rats), ifenprodil (0.2 μg in 1 μl PBS, n = 8 rats), or Ro25-6981 (5.0 μg in 1 μl PBS, n = 6 rats) was infused into the CA1 region 15 min before memory retention was tested. PBS was similarly infused as vehicle control (1 μl, n = 9 rats). As shown in Figure 7A1, An one-way ANOVA revealed a significant group effect on contextual freezing scores (F(4,31) = 8.12, *p *< 0.05). Planned comparison showed that the rats treated with NVP-AAM077, ifenprodil, or Ro25-6981 exhibited a deficient contextual fear memory (F(1,13) = 15.48, *p *< 0.01 for 0.012 μg NVP-AAM077 vs. vehicle; F(1,14) = 10.61, *p *< 0.01 for 0.12 μg NVP-AAM077 vs. vehicle; F(1,15) = 20.71, *p *< 0.01 for ifenprodil vs. vehicle; F(1, 13) = 13.23, *p *< 0.01 for Ro25-6981 vs. vehicle). However, each group of rats demonstrated a normal auditory fear memory (Figure 7A2: F(4, 21) = 0.62, *p *> 0.05).

Then, we examined the effects of intra-CA1 inhibition of NR2B- as well as NR2A-NMDARs on memory retrieval for the five CS-US pairing conditioning. NVP-AAM077 (0.012 μg in 1 μl PBS, n = 6 rats), ifenprodil (0.2 μg in 1 μl PBS, n = 6 rats), or Ro25-6981 (5.0 μg in 1 μl PBS, n = 5 rats) was infused into the CA1 region 15 min before memory retention test. PBS was similarly injected as vehicle control (1 μl, n = 7 rats). As shown in Figure 7B1, an one-way ANOVA revealed a significant group effect on contextual freezing scores (F(3,20) = 8.12, *p *< 0.01). Planned comparison showed that the rats treated with NVP-AAM077, ifenprodil, or Ro25-6981 demonstrated a deficient contextual fear memory (F(1,11) = 19.66, *p *< 0.01 for NVP-AAM077 vs. vehicle; F(1,11) = 12.10, *p *< 0.01 for ifenprodil vs. vehicle; F(1, 10) = 13.06, *p *< 0.01 for Ro25-6981 vs. vehicle). Auditory fear memory was intact upon the drug infusion (Figure 7B2: F(3,16) = 0.22, *p *> 0.05).

Taken together, these results strongly suggest that NR2B-NMDARs, as well as NR2A-NMDARs, in the CA1 region are required for the retrieval of contextual fear memory induced by the one and five CS-US pairing protocols.

## Discussion

The present study shows that NR2B-NMDARs are required for LTP induced by the spike-timing protocol, but not for LTP induced by the pairing or HFS protocol. Intracellular Ca^2+ ^signals triggered by spiking-timing and pairing protocols display different kinetics. Late-phase LTP induced by HFS protocol does not involve NR2B-NMDARs. Moreover, the significance of NR2B-NMDARs in acquisition of contextual fear memory is dependent on conditioning protocols.

### Role of NR2B-NMDARs in LTP

Many previous studies using different experimental approaches have shown that NR2B-NMDARs contribute to synaptic potentiation in different areas of the central nervous system [12-14,24-26]. However, some other studies have reported that NR2B-NMDARs are not required for synaptic potentiation or LTP [9,10]. Considering that NR2B- and NR2A- NMDARs have different biophysical properties and couple to different intracellular signaling cascades [8,26-29], it may be possible that different induction protocols may activate different NMDAR subtypes. Indeed, it has been reported that different LTP-inducing protocols recruit different signaling pathways. For example, in the amygdala, pairing-protocol induced LTP depends on L-type voltage-gated calcium channels (L-VGCCs) but not NMDARs, while tetanus-stimulation induced LTP involves NMDARs but not L-VGCCs [30]. In the present study, we demonstrate that the involvement of NR2B-NMDARs in LTP is dependent on induction protocols. NR2B-NMDARs are required for LTP induced by the spike-timing protocol, but not by the pairing protocol. Although it has been reported that a low dose Ro25-6981 (0.3 μM) enhances, but not reduces NMDAR-mediated EPSCs in the CA1 region [24,31], the same dose Ro25-6981 in the present study reduced LTP induced by the spike timing protocol, but not by the pairing protocol (Table 2).

**Table 2:** Effects of Ro25-6981 on LTP induced by different protocols in the anterior cingulate cortex (ACC) and hippocampal CA1 region

**Induction Protocol**	**ACC (Zhao et al., 2005)**	**CA1 (the present study)**
*Spike timing protocol*	LTP blocked	LTP reduced or blocked
*Pairing protocol*	LTP reduced	Normal LTP
*HFS protocol*	--	Normal LTP

The induction of spike timing-dependent LTP requires activation of NMDAR [20,21]. Spike timing-dependent potentiation requires the temporal window between presynaptic spikes and postsynaptic EPSPs. It is known that NR2B- and NR2A-NMDARs have different characteristics: NR2B-NMDARs have slower kinetics than NR2A-NMDARs [27]. It is possible that NR2B-NMDARs are more sensitive to the certain form of LTP induction protocol. Berberich et al. reported that the amount of charge transfer during LTP induction is a critical factor in low-frequency stimulation pairing [31]. Thus, there is such a possibility, although remains to be demosntrated, that inhibition of NR2B-NMDARs affect the charge transfer during LTP induction.

The induction-protocol dependent involvement of NR2B-NMDARs in LTP suggests that LTP induced by different protocols may have different intracellular mechanisms. Indeed, it has been suggested that different LTP induction protocols may activate distinct signaling cascades that generate LTP with different expression mechanisms [32-34]. LTP induction in the CA1 region requires Ca^2+ ^influx through NMDAR, either through direct activation of Ca^2+^-sensitive substrates and/or subsequent Ca^2+ ^release from intracellular Ca^2+ ^stores [1,35]. Ca^2+ ^influx activates calcium/calmodulin-dependent protein kinase II (CaMKII), which is required as a critical cellular cascades for NMDA-dependent LTP [11,36]. Recently, Gerkin et al. reported that NMDAR subtypes can differentially activate competitive signaling modules in the induction and integration of spike-timing-dependent plasticity [37]. That is, Ca^2+ ^influx via NR2B-NMDARs may activate either CaMKII or calcineurin (CaN), which is dependent on the outcome of dynamic competition driven by NMDA receptor subtypes. Our data suggested that Ca^2+ ^influx via NR2B-NMDARs induced by spike-timing protocol can activate CaMKII and lead to LTP. Furthermore, our calcium imaging in the present study showed that the NR2B-NMDAR mediated Ca^2+ ^transients were faster under the spike-timing than pairing protocols, which might explain the different significance of NR2B-NMDARs in LTP under the two protocols since fast Ca^2+ ^transients are better for LTP, but slow Ca^2+ ^transients not [38].

### Role of NR2B-NMDARs in memory

It has been documented that NR2B-NMDARs in the hippocampus, amygdala and anterior cingulate cortex play an important role in memory or persistent pain [14,24,39,40]. Genetic over-expression of NR2B receptor subtype in the forebrain enhances spatial memory as well as contextual fear memory [14], whereas pharmacological blockade of NMDARs in the CA1 region impairs contextual fear memory [19]. Pharmacological blockade of NR2B-NMDARs in the lateral amygdala or in the ACC impairs formation of fear memory [23,24]. However, it is unclear whether hippocampal NR2A- and NR2B-NMDARs have differential roles in fear memory formation.

The present study shows that, unlike NR2A-NMDARs that are required non-differentially, NR2B-NMDARs are involved in acquisition of contextual fear memory in a conditioning-strength dependent way. Pre-conditioning intra-CA1 infusion of the NR2B selective antagonist ifenprodil or Ro25-6981 impaired contextual fear memory induced by five but not one CS-US pairing protocol, while similar treatment with the NR2A antagonist NVP-AAM077 disrupted memory for both protocols. This is well consistent with a recent study in our laboratory [41], showing that NR2B-NMDARs in the basolateral nucleus of amygdala (BLA) are involved in acquisition of auditory fear memory also in a conditioning-strength dependent way. In that study, we reported that pre-conditioning intra-BLA infusion of the NR2B selective antagonist ifenprodil or Ro25-6981 impaired auditory fear memory induced by five but not one CS-US pairing protocol, while similar treatment with the NR2A antagonist NVP-AAM077 interfered with memory for both protocols. Consistently, genetic over-expression of NR2B C-terminal in the BLA, which interferes with the C-terminal mediated intracellular signaling, produced a severe deficit in auditory fear memory for five but not one CS-US pairing protocol, whereas over-expression of NR2A C-terminal produced a deficient memory for both protocols. There seems a recruitment mechanism in the hippocampus and amygdala for involvement of NR2B-NMDARs in memory acquisition: the heavier the training load is, the more involved the NR2B-NMDAR is.

### Selectivity of NVP-AAM077

NR2A- and NR2B-NMDARs have distinct electrophysiological and signaling properties [8,27,42-48]. Dissection of the functions of NR2A- and NR2B-NMDARs will promote our understanding of their roles in learning and memory. As specific antagonists for NR2A-NMDAR are not available, the role of NR2A-NMDARs in learning and memory is still poorly understood. Recently, a relatively selective NR2A-NMDAR antagonist, NVP-AAM077, has been developed. However, some previous studies argue that NVP-AAM077 is not sufficient to discriminate between NR2A- and NR2B-NMDARs, with about 10-fold higher selectivity for NR2A- than for NR2B-NMDARs [49,50].

In the present study, we used NVP-AAM077 (0.012 or 0.12 μg in 1.0 μl vehicle) for pharmacological blockade of NR2A-NMDARs in the CA1 region. We found that treatment with NVP-AAM077 non-differentially impaired the acquisition of fear memory induced by the two conditioning protocols, one of which included a single CS-US pairing and the other five CS-US pairings. Although NVP-AAM077 could act at NR2B as well, it is unlikely that the NVP-AAM077 effect was mediated by NR2B-NMDARs, because similar treatment with the selective NR2B-NMDAR antagonist ifenprodil (0.2 μg in 1.0 μl vehicle, equal molarity with 0.12 μg/1.0 μl NVP-AAM077) or Ro25-6981 (5.0 μg) impaired the acquisition of fear memory induced by the single CS-US pairing protocol. This result strongly suggests an essential role of NR2A-NMDARs in the CA1 region in acquisition of contextual fear memory, regardless of conditioning strength.

In summary, the present study strongly suggests an induction- and conditioning-protocol dependent involvement of NR2B-NMDARs in the CA1 region in long-term potentiation and contextual fear memory formation.

## Competing interests

The authors declare that they have no competing interests.

## Authors' contributions

X–HZ, L–JW, BG and MR carried out the electrophysiological studies. X–HZ also performed the behavior experiments. L–JW performed Ca imaging experiments. B–ML and MZ conceived of the study, and participated in its design and coordination. All authors read and approved the final manuscript.
